# Kinetics of lung tissue factor expression and procoagulant activity in bleomycin induced acute lung injury

**DOI:** 10.1186/s40169-015-0063-4

**Published:** 2015-06-21

**Authors:** Li Ma, Ciara M. Shaver, Brandon S. Grove, Daphne B. Mitchell, Nancy E. Wickersham, Robert H. Carnahan, Tracy L. Cooper, Brittany E. Brake, Lorraine B. Ware, Julie A. Bastarache

**Affiliations:** Division of Emergency Intensive Care Unit, the Second Hospital of Lanzhou University, Lanzhou, China; Division of Allergy, Pulmonary, and Critical Care Medicine, Vanderbilt University School of Medicine, Nashville, USA; Department of Cancer Biology, Vanderbilt University School of Medicine, Nashville, USA; Vanderbilt Institute for Chemical Biology, Nashville, USA; Department of Pathology, Microbiology and Immunology, Vanderbilt University School of Medicine, Nashville, USA

**Keywords:** Acute lung injury, Acute respiratory distress syndrome, KC, Inflammation, Coagulation, Permeability, Pulmonary edema

## Abstract

**Background:**

Activation of coagulation by expression of tissue factor (TF) in the airspace is a hallmark of acute lung injury (ALI) but the timing of TF activation in relationship to increases in lung permeability and inflammation are unknown.

**Methods:**

To test the hypothesis that TF is upregulated early in the course of acute bleomycin lung injury and precedes increased permeability and inflammation we studied the early course of bleomycin-induced ALI in mice. Mice were treated with 0.04U intratracheal bleomycin or vehicle control and bronchoalveolar lavage (BAL) and lung tissue were collected daily for 7 days. Whole lung TF mRNA was determined by QT-PCR. TF protein was assessed by ELISA and immunostaining. BAL procoagulant activity was measured by BAL clot time and thrombin-antithrombin complexes. Inflammation was assessed by BAL cell count, differentials and CXCL1/KC concentration. Lung permeability was assessed by BAL protein and lung wet to dry weight ratio.

**Results:**

Expression of CXCL1 occurred by day 1. BAL protein and lung wet-to-dry weight ratio increased significantly by day 3. TF mRNA and BAL procoagulant activity peaked on day 4 while whole lung TF protein peaked on day 6. Changes in permeability and procoagulant activity preceded inflammatory cell influx which was maximal at day 6 while whole lung TF protein peaked along with inflammation.

**Conclusion:**

These data demonstrate that cytokine upregulation is the earliest response to bleomycin administration, followed by increased lung permeability, upregulation of TF, and recruitment of inflammatory cells.

## Background

Acute lung injury (ALI) and Acute Respiratory Distress Syndrome are caused by local or systemic insults and involves loss of alveolar-capillary barrier integrity with influx of protein-rich edema fluid into the airspace, migration of inflammatory cells such as neutrophils and macrophages into the affected lung [1, 2] and activation of procoagulant pathways leading to intra-alveolar thrombin formation and fibrin deposition [3–7]. Despite the fact that these hallmark features of ALI are well described, the specific timing of increased permeability, influx of inflammatory cells and activation of coagulation are unknown. Given the lack of specific therapies for ALI, a detailed understanding of the kinetics of activation of coagulation pathways and the relationship to other indices of lung injury is particularly important so that coagulation-targeted therapies may be administered at the correct time in the course of injury.

Inflammatory cell influx into the airspace has been well studied. In mice, one of the major chemotactic factors contributing to neutrophil recruitment to the lung is CXCL1, an early proinflammatory chemokine that plays a key role in recruitment of inflammatory cells [8], Although animal studies aimed at preventing neutrophil recruitment to the lung have been promising [9], there have not been any major breakthroughs in the development of new therapeutics for human ALI [2]. Inflammation and coagulation are tightly linked processes with inflammatory cytokines leading to upregulation of Tissue Factor (TF) on lung epithelial cells [10], macrophages [11, 12] and endothelial cells [13]. Studies suggest that generation of thrombin as a result of TF activation leads to inflammatory cell recruitment. Coagulation and inflammation are linked through protease activated receptors (PAR) and thrombin-mediated signaling through PAR-1 on vascular cells increases production of chemokines such as MCP-1, resulting in the efficient recruitment of leukocytes in a mouse-to-rat heart transplant model [14]. These studies suggest that targeting coagulation pathways in ALI may lead to changes in inflammation. Thus a more detailed understanding of the temporal relationship between coagulation and inflammation is needed to identify the underlying molecular mechanisms that regulate inflammation and may lead to the development of novel therapeutic strategies in ALI.

In recent years numerous studies have focused on the role of coagulation pathways in the pathophysiology of ALI/ARDS [6, 7, 10, 15–19]. Among them, TF, the principal initiator of the extrinsic coagulation pathway has been the focus of several studies. Activation of the extrinsic coagulation cascade through up-regulation of TF specifically in the lung epithelium has been implicated in the pathogenesis of acute lung injury [10, 20] in humans and animal models [21–25]. We hypothesized that upregulation of lung epithelial TF would occur early in the course of bleomycin injury and would precede inflammatory cell influx. Using the bleomycin-induced acute lung injury model which causes intense lung epithelial injury, a major source of TF in the lung [10], we measured indices of TF upregulation and activation, lung permeability and lung inflammation. The current study focuses on the temporal relationship of multiple aspects of ARDS pathogenesis. While prior animal studies have examined TF expression in response to bleomycin, these studies were focused either on later time points when bleomycin-induced fibrosis is established or on a single aspect of lung injury. In the current study we set out to comprehensively study lung TF regulation, permeability and inflammation in the early inflammatory stages of bleomycin-induced lung injury, something that has not been previously reported. We found that expression of the chemokine CXCL1 occurred by day 1 after IT bleomycin. This was followed by maximal upregulation of TF mRNA and procoagulant activity at day 3 which correlated with increased permeability. Maximal inflammatory cell influx and TF protein expression occurred later at day 6. Taken together, our findings indicate that increased permeability and upregulation of TF in the lung occur early in the course of bleomycin-induced ALI and that these changes precede inflammatory cell recruitment to the lung.

## Methods

### Mouse experimental protocol and tissue collection

All the animal experiments were conducted in accordance with the institutional guidelines and were approved by the Vanderbilt Institute for Animal Care and Use Committee. Eight to 10-week-old male and female wild-type (WT) C57Bl/6 mice were anesthetized with isofluance and given a single direct intra-tracheal (IT) injection of 100uL of 0.04 U bleomycin sulfate (Bristol-Myers Squibb Co., Princeton, NJ) or sterile saline (control) as previously described [21]. Six to 10 mice per group were studied daily on days 1–7. At each time point bleomycin-treated and control mice were sacrificed for BAL and tissue collection. In separate experiments, lung tissue was collected for immunostaining and wet to dry weight ratio. For each measurement, all available specimens were assayed.

### Bronchoalveolar lavage cell count and differential

BAL samples were collected by instilling 900 μl 0.9 % NaCl and aspirating the fluid. BAL was centrifuged at 1000xg for 10 min and supernatant was frozen at −80 °C. Manual cell counts (hemocytometer) and cytospins/differentials were completed with fresh BAL fluid. Cytospins were stained with Hema 3 staining kit (Thermo-Fisher Scientific, Pittsburg, PA, USA) [21].

### Isolation and analysis of TF mRNAs

RNA was extracted following the commercial protocol with an Invitrogen Pure Link RNA mini kit and quantified by absorbance at 260 nm. mRNA (1 μg) was used to synthesize first-strand cDNA with a SuperScript VILO cDNA kit (Invitrogen). Quantitative real-time PCR was performed using Assays-on-Demand primer/MGB probes (TaqMan probe/primer sets, Applied Biosystems) for murine TF (Mm00438853) and GAPDH (Mm99999915). All values were normalized to the GAPDH mRNA content of each sample.

### Murine TF quantification by ELISA

To quantify TF protein in whole lung homogenate, we developed a novel murine TF ELISA. General ELISA processing and procedures for assay development was performed as previously described [26]. All lung homogenate test samples were assayed in duplicate. Standard curves for quantitation of TF levels within the homogenates were generated by spiking known quantities of purified recombinant mouse TF (R&D systems) into each well which was captured utilizing a rat monoclonal anti-TF antibody (generously provided by Daniel Kirchofer, Genentech, San Francisco, CA) and detected utilizing a goat polyclonal anti-TF antibody (R&D systems). As initial experiments demonstrated that quantitation of known TF levels in standard assay buffer supplemented with 1 % BSA performed equivalently to parallel experiments in lung tissue homogenate, 1 % BSA was utilized as the sample matrix for all standards.

### BAL TATc and CXCL1 concentration by ELISA

TATc (Enzygnost® TAT micro kit, Siemens, Tarrytown, NY) and CXCL1 (DuoSet, R&D Systems, Minneapolis, MN) were quantified by ELISA according to manufacturers instructions.

### Clot time measurement

Clot time was measured using a mechanical clot detection system (STart4 Coagulometer; Diagnostica Stago, Paris, France) according to published methods [21]. Briefly, 25 μl of BAL was warmed for 15 min at 37 °C then incubated with 25 μl of pooled citrated mouse plasma (Bioreclamation, East Meadow, New York). Clot time was determined in duplicate as plasma recalcification time following the addition of 25 μl of 50 mM calcium chloride.

### BAL protein measurements

Protein was measured in BAL using the BCA protein assay (Pierce, Rockford, Illinois, USA).

### Lung TF immunostaining

Formalin-fixed and paraffin-embedded lung sections from day 1, day 3, day 5 and day 7 were immunostained for TF. Lung sections were de-paraffinised with antigen retrieval by standard procedures as previously described [21]. Slides were incubated overnight at 4 °C with 1: 200 goat polyclonal anti-TF Ab (R&D Systems), rinsed and incubated with a biotinylated rabbit anti-goat secondary antibody (Innogenex, San Ramon, California) for 20 min at room temperature. Slides were developed with NovoRed (Vector, Burlingame, California) for 30s and counterstained with methyl green for 10s.

### Lung wet-to-dry weight ratio

The left lung was placed on pre-weighed aluminum foil, weighed and placed in an 80 °C oven for 2 days. The foil + lung was weighed again and the ratio was calculated ((lung − foil before drying)/(lung − foil after drying)).

### Statistical analysis

Normally distributed data are displayed as bar graphs showing mean and SEM. Comparison of three or more groups was done using one-way analysis of variance with post hoc Tukey test. Comparison of the two groups was by Student’s *t* test. All analyses were done with SPSS software version 22 for Macintosh. *p* < 0.05 was considered statistically significant.

## Results

### TF regulation in the time course of bleomycin-induced ALI

**Fig. 1:**
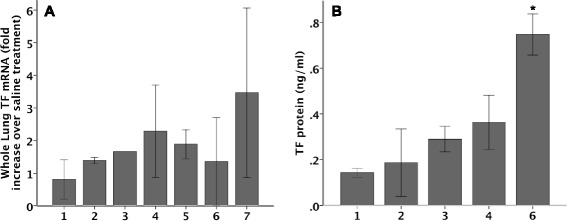
TF mRNA and protein expression in whole lung over time. TF mRNA (**a**) and protein (**b**) were measured in whole lung from bleomycin treated mice. There was a non-significant increase in TF mRNA starting at day 2 with the peak at day 4. The right panel shows TF protein concentration in lung homogenate as measured by TF ELISA. *N* = 2-5 per group. *p < 0.007 versus all other groups by ANOVA with post-hoc Tukey test

**Fig. 2:**
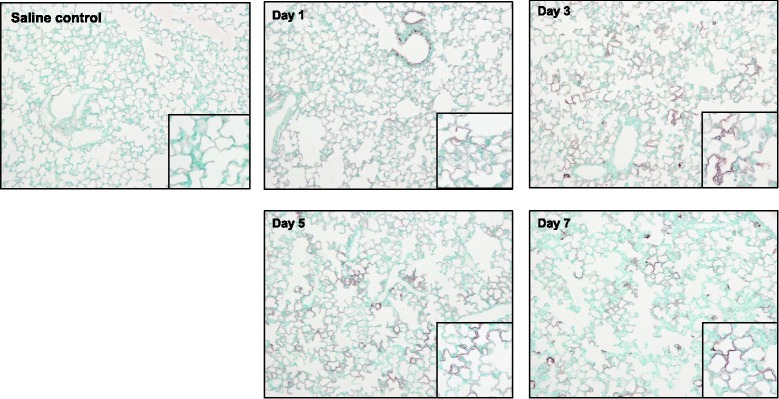
TF protein localization in lungs following IT Bleomycin. TF protein expression in mouse lung measured by TF immunostaining by days after bleomycin treatment. A saline control treated animal is shown in the left panel for comparison. Larger images at 10x power, insets at 20x power

**Fig. 3:**
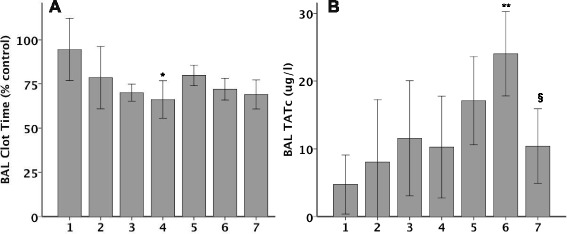
BAL procoagulant activity over time. TF procoagulant activity was measured by BAL clot time (Panel **a**) and BAL thrombin-antithrombin complexes (Panel **b**) over time in response to IT bleomycin. Clot time is expressed as percent of the saline-treated animals on the same day. *N* = 5-6 per group. *p = 0.024 versus day 1 **p < 0.001 and §*p* = 0.028 versus PBS control by t test on each day

In order to define the time course of TF regulation we measured TF mRNA and protein (Fig. 1), cellular localization by immunostaining (Fig. 2) and activity (Fig. 3) over time in response to IT bleomycin treatment. In the bleomycin treated group, TF mRNA increased slightly starting at day 2 with peaks at day 4 (Fig. 1a). TF protein in whole lung homogenates as measured by ELISA (normalized to total protein) increased daily and peaked at day 6 (Fig. 1b). Increased TF protein expression in the lung epithelium was evident by day 1 by TF immunostaining (Fig. 2). Staining became more patchy and intense on days 3 and 5 and persisted on day 7. These increases in TF mRNA and protein were paralleled by increased TF procoagulant activity in BAL fluid (Fig. 3a) which also peaked at day 4. Consistent with TF procoagulant activity and generation of thrombin, thrombin anti-thrombin complexes started to increase at day 3 and peak on day 6 (Fig. 3b).

### C57BL/6 mice have increased pulmonary edema in response to intra-tracheal bleomycin

**Fig. 4:**
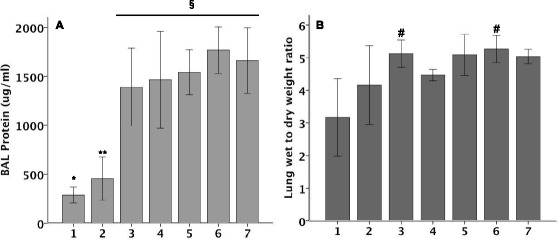
BAL protein (Panel **a**) and wet to dry lung weight ratio (Panel **b**) over time on mice treated with IT Bleomycin. *N* = 5-6 per group. *p = 0.006, **p = 0.017, §*p* < 0.001, #*p* < 0.05 versus PBS treatment by t test

Bleomycin treated mice had increased BAL protein concentration, an indicator of lung vascular permeability. Compared to day 1, BAL protein levels were 2- to 4-fold higher on days 3–7 (Fig. 4a) suggesting that increased permeability in response to IT bleomycin persists for the first week after injury. Similarly, mice had significant increases in wet-to-dry weight ratio by day 3 following treatment with IT bleomycin (Fig. 4b). This increase in excess lung water persisted out to 7 days.

### Inflammatory response to IT bleomycin

**Fig. 5:**
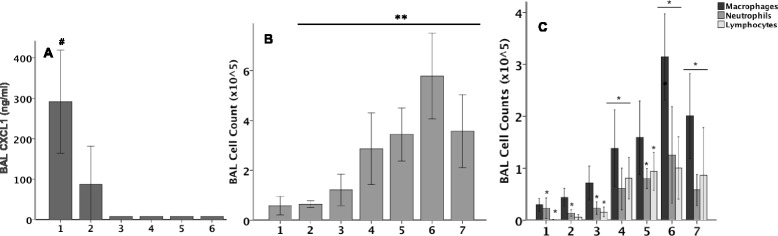
Lung inflammation following IT bleomycin. BAL CXCL1 (Panel **a**) total cell count (Panel **b**) and differential cell counts (Panel **c**) over time in response to IT Bleomycin. While CXCL1 levels peak at day 1, BAL inflammatory cells peak at day 6. *N* = 5-6 per group. #*p* < 0.05 versus PBS, **p < 0.05 PBS, *p < 0.05 PBS all by t test

In order to investigate the early inflammatory response following IT bleomycin, mice were studied daily for 7 days with BAL cell counts with differentials and CXCL1 levels. BAL CXCL1 levels peaked at day 1 and rapidly declined (Fig. 5a). BAL cell counts started to increase by day 3 but peaked on day 6 (Fig. 5b). The majority of inflammatory cells were macrophages at all time points (Fig. 5c) but there were also significant increases in neutrophils (day 6) and lymphocytes (days 4–7).

## Discussion

Acute lung injury and its most severe form, acute respiratory distress syndrome (ARDS) are marked by severe hypoxemia, diffuse alveolar damage, profound influx of activated inflammatory cells into alveoli, along with protein-rich pulmonary edema accumulation that impairs lung function [27]. As mechanisms of ALI pathophysiology have been elucidated with time, other important pathways that mediate the lung injury response have been identified including activation of coagulation, specifically by upregulation of TF on the lung epithelium [10, 15]. Among these factors involved in ALI pathophysiology, neutrophil migration is a hallmark of ALI and is induced through a complex interplay of adhesion molecules, cytokines and chemokines [28]. Another hallmark of ALI is increased alveolar-capillary permeability leading to influx of protein rich edema fluid into the airspace. The mechanisms that regulate permeability have been studied and include thrombin activation and fibrin deposition that enhance lung inflammation, activate endothelial cells and disrupt lung paracellular permeability [29]. Others have shown that TF is upregulated in the lung in response to bleomycin-induced lung injury [22–25, 30] and that procoagulant pathways are important in modulating the development of pulmonary fibrosis following an acute insult. Our prior work has focused on regulation of coagulation proteins by the lung epithelium. In ALI, the lung epithelium upregulates TF mRNA, protein and procoagulant activity [10] and releases TF positive procoagulant microparticles [15] leading to a shift towards procoagulant pathways in the airspace that are not balanced by anticoagulant pathways such as tissue factor pathway inhibitor (TFPI) [16]. While other important studies have established a critical role for TF in modulating lung coagulation, inflammation, permeability and fibrosis in the later stages of bleomycin induced lung injury (7–28 days), relatively little is known about the relationship between TF and ALI in very early bleomycin induced lung injury.

In this study we define the time course of TF expression, lung permeability and inflammation in a direct model of bleomycin-induced acute lung injury. Because the lung epithelium is a major source of TF in the airspace, we chose a model of ALI, intratracheal bleomycin, that causes significant lung epithelial injury. While our prior studies do establish that the lung epithelium is a major source of TF, the relationship between TF and lung permeability and inflammation in the early stages of ALI are unknown.

In this study we define the kinetics of TF expression in the lung at the mRNA, protein and activity level. We were surprised to find that the peak in total lung TF expression at day 6 occurred after the peak in procoagulant activity as measured by clot time. There are several possible explanations for this finding. First, it may be that the early upregulation of TF in the lung results in the release of large numbers of TF containing procoagulant microparticles (MPs). We have previously shown that there are high concentrations of TF containing MPs in the airspace in ARDS [15]. Our measurements of TF by ELISA were done on lungs that had been lavaged (BAL) prior to snap freezing. It is possible that this lavage removed TF containing MPs that had been released by the lung epithelium and thus our ELISA data do not represent the total amount of TF generated by the lung. BAL will also remove alveolar macrophages, another potential source of TF in the lung [31]. Another potential explanation for the differences in timing of TF mRNA, protein and procoagulant activity is that although TF transcription is not increasing from days 4–7, TF translation may be increased resulting in more TF protein. We did not study TF translation in these experiments so this remains an unanswered question. Other groups have shown increases in TF mRNA in response to bleomycin while our increases were modest and not statistically significant. Olman et al. showed a 2.3-fold increase by day 1 and a 3.8-fold increase by day 4 in TF mRNA [24]. One potential explanation for this discrepancy is that our study was underpowered to find a statistically significant difference in mRNA. There may also be differences related to measurement technique and bleomycin dose (higher in the Olman study). Alternatively, TF mRNA may be increased in a selected cell population, such as the lung epithelium, which may be obscured by measuring TF mRNA in the whole lung. Finally, we found elevated TATc levels suggest that active TF results in downstream activation of the coagulation cascade. It is well known that the concentration of TATc in blood reflects the formation of thrombin [32, 33] and serves as a sensitive marker for the activation of coagulation [34, 35]. In the bleomycin group the increase in TAT level provided evidence of a downstream activation of the coagulation pathway in the airspace in the early stage of ALI.

In the current study, we show that increased BAL CXCL1 is the earliest change in the lung (day 1) followed by increase permeability (days 3–7) and increased TF mRNA and procoagulant activity by day 4. All of these changes precede the influx of inflammatory cells which peak at day 6. CXCL1 is known to be important in neutrophil recruitment in bleomycin-induced acute lung injury. Russo et al. showed an early increase in lung tissue CXCL1 6 hours after bleomycin that persisted through day 4 [36]. Interestingly, this group also showed that BAL neutrophil influx was delayed, peaking at day 8. This was similar to our findings of CXCL1 increase on day 1 followed by neutrophil influx on day 6. Whether other chemokines drive neutrophil influx into the airspace in the bleomycin model remains to be seen. We were surprised to find that changes in TF and permeability persistent through day 7 despite reduced inflammation by this time point. One explanation for this finding is that mechanisms other than inflammation and coagulation affect disruption of the alveolar-capillary barrier. However, an alternative possibility is that a secondary lung insult, such as infection, may have developed within the first week. Further work is needed to elucidate mechanisms affecting prolonged permeability disruption after bleomycin.

One surprising finding is that the permeability changes occurred early and preceded increases in TF or TATc formation. This suggests the possibility that influx of mediators from the plasma into the airspace are necessary either for upregulation of lung TF or for activation of downstream coagulation. Although it is well described by our group and others that TF protein and activity is regulated locally in the lung epithelium [10, 20], whether resident lung cells can make other necessary coagulation factors such as factors VII, X, prothrombin and fibrinogen is unknown. It is possible that although the lung epithelium can upregulate TF, proteins from the circulation are needed for propagation of coagulation and generation of thrombin and fibrin. Our study as designed cannot definitively answer this question but the temporal relationship between increased permeability and activation of coagulation suggests this as one possibility. This study adds to the growing body of literature supporting an integral role for TF in mediating lung injury, permeability and inflammation.

There are some limitations to this study. First of all, we only studied one chemokine, CXCL1, in the time course but there are other chemokines and cytokines that may play important roles. Prior studies of bleomycin-induced ALI have demonstrated that interleukin (IL)-1β, IL-6, IL-8, and MIP-2a/CXCL2a, which are associated with inflammation are increased in bleomycin induced lung injury [37, 38]. Second, we found that BAL inflammatory cells were maximal at day 6 which occurred after maximal coagulation activation. This suggests TF may be important in the recruitment of inflammatory cells to lung but how TF upregulation in the lung may influence inflammatory cell recruitment to the airspace is unknown and requires further study. Finally, our study is descriptive in nature although it provides critical information of the timing of TF regulation and activation in relationship to permeability and inflammation in the lung.

## Conclusions

In this study of kinetics of TF, permeability and inflammation in early bleomycin induced lung injury, we found that permeability changes precede upregulation in TF supporting the concept that therapies aimed at limiting alveolar capillary permeability may have the potential to influence regulation and activation of TF. Likewise, the fact that TF activity peaks prior to the peak in inflammatory cell influx suggest that strategies aimed at modulating TF activity have the potential to influence inflammation in the lung. Future studies will build on these important findings to test the therapeutic potential of modulating TF in the lung in ALI.
